# Usefulness of Tilt Testing in Children with Syncope: A Survey of Pediatric Electrophysiologists

**Published:** 2008-11-01

**Authors:** Anjan S Batra, Seshadri Balaji

**Affiliations:** 1 University of California-Irvine, CA; 2 Oregon Health Sciences University, Portland, OR

**Keywords:** syncope, diagnostic procedure, electrophysiology

## Abstract

The role of tilt table testing as a diagnostic modality in children with unexplained syncope is unclear.  We sent a questionnaire to members of the Pediatric and Congenital Electrophysiology Society to assess the current practice pattern. Of the 186 members, 97 (52%) replied.  Twenty four percent of the pediatric electrophysiologists have completely stopped doing tilt table tests and of those performing the tests, a majority (76%) did < 10 tests/yr (median=3 tilts/yr, range 0-100/yr).  Of those performing the test, 95% rarely or never accepted direct referrals from the general practioners and 62% felt that the frequency of tilt table tests being performed had decreased since they had started practicing. The median usefulness of the test was rated at 3 (range 1-9) on a scale of 1 to 10 with 10 being very useful. A majority (68%) felt they rarely or never altered treatment based on the results of the tilt test.  Wide variability was noted in the test protocol including the tilt angle, tilt duration, use of pharmacologic agents and the duration of fasting prior to the test. We therefore conclude that there is significant lack of standardization in tilt table tests performed in children. Tilt table testing, as perceived by pediatric electrophysiologists, is of limited utility and progressively less used in children with syncope.

## Introduction

Syncope is defined as a transient loss of consciousness and postural tone due to inadequate cerebral circulation. It is a common and often frustrating clinical problem encountered by pediatricians in outpatient or emergency room settings. Neurocardiogenic syncope is believed to be the most common cause of syncope in the absence of structural heart disease [1-3].  It is estimated that up to 15% of children will experience a syncopal episode before the end of adolescence [4].

Historically, the determination of neurocardiogenic syncope was made in the presence of a classic clinical history and after the exclusion of other causes of syncope. The use of tilt table testing for evaluation of syncope became widely popular in the 1990s after the publication of several key studies [5-8]. The American College of Cardiology published guidelines in 1996 on the indications of tilt table testing [9]. The general agreement was that tilt table testing should be done in patients with recurrent syncope or in high risk patients after a single syncopal episode. These guidelines were based on studies done mostly in adults [2].

Initial studies in children found the head-up tilt test to be a useful tool for investigating neurally mediated syncope [10-12]. Over the last decade, however, the utility of tilt table testing for evaluation of pediatric syncope has been increasingly questioned [13-17]. As pediatric electrophysiologists (EPs) are the most likely subset of physicians dealing with syncope in children, we designed a questionnaire directed at them to better understand the current practice of tilt table testing in patients with syncope.

## Materials and Methods

We sent a 24-question survey to physician members of the Pediatric and Congenital Electrophysiology Society (PACES) to assess the current practice pattern. These 24 questions addressed issues regarding the frequency, trends, and methods and perceived utility of tilt table tests (Table 1). Individual physicians, rather than institutions were surveyed. The numbers in the results section are based on estimates reported by individual physicians. The study was approved by the institutional review board at the University of California-Irvine and by the executive board of PACES. All values are expressed as medians. Statistical analyses were done with paired t tests. Significance was accepted at P ≤ 0.05.

## Results

Of the 186 PACES members worldwide, 97 (52%) participated in the study. The pediatric EPs responding had been in practice for a median of 12 years (range 1 - 27 years) and practiced in groups with a median of 10 cardiologists (range 1-45).  Twenty four percent of the pediatric EPs have completely stopped doing tilt table tests and of those performing the tests, a majority (76%) did < 10 tests/yr (median=3 tilts/yr, range 0-100/yr).  Of those performing the test, 95% rarely or never accepted direct referrals from the general practioners for tilt table tests and 62% felt that the frequency of tilt table tests being performed had decreased since they had started practicing.

Medications used to enhance the sensitivity of the test included isoproterenol (79%), nitroglycerine (5%) and other miscellaneous drugs (16%). Those who performed the test used a variety of tilt angles [60° (24%), 70° (36%), 80° (36%), 90° (1%), and variable (3%)]. Likewise, there was wide variation in the duration of tilting before declaring it negative [< 10 min (4%), 10-15 min (14%), 16-20 min (26%), 21-25 min (18%), >25 min (36%) variable (2%)]. The duration of fasting (nothing by mouth status) before the test was also variable [< 2 hours (18%), 2-4 hours (47%), > 4 hours (19%), variable (16%)].  The median usefulness of the test was rated at 3 (range 1-9) on a scale of 1 to 10 with 10 being very useful. At least one major shortcoming was reported by 97% of the physicians.  These included a low sensitivity (70%), a long time for test (37%), low reimbursement (21%), patient discomfort (26%), a low specificity (21%), and no added benefit to clinical history (10%).  A majority (68%) felt they rarely or never altered treatment based on the results of the tilt test. There was no significant difference in the number of tests per physician over the last 12 months between the small (median: 2; range 0-80), medium (median: 4, range 0-100) and large institutions (median: 3, range 0-100) (p=ns); between the academic (median: 4, range 0-100) and private practice models (median: 2.5, range 0-80) (p=ns); or between different regions within and outside the United States.

## Discussion

The main findings of our survey were that relatively few tilt table tests are being done by pediatric EPs and that the majority feels that the frequency of tilt table tests is decreasing in their practice. The reasons for these perceptions are unclear but are likely influenced by the perceived low usefulness of the test and also the feeling that tilt table testing has numerous shortcomings, a notion expressed by almost all of the respondents.

We also found wide variation in the testing protocols with an absence of consensus on tilt angle, tilt duration, use of pharmacologic agents during the test, and whether patients need to be fasting before a test, and if so, for how long. Again the reasons for these differences in protocol are unclear.

Although 95% of the physicians chose a tilt angle between 60 and 80 degrees, there was no consensus on a more specific tilt angle within this range.  The positivity rate has been shown to be higher at steeper angles (80 vs. 60 degrees) [15,17].  In addition, children appear to be more susceptible to orthostatic stress than adults and symptoms of pre-syncope and frank syncope may be elicited in up to 60% of normal control teenaged volunteers when tilted at an angle of 80 degrees [16].

The duration of upright posture is probably the most critical determinant of the sensitivity and specificity of the tilt test [1]. A significant proportion of physicians favored a shorter duration of upright posture in children, which is in contrast to most published reports in adults that have tended to favor relatively long drug free initial tilt duration of 45 minutes [18]. Although the longer periods may increase the sensitivity of the test, they may be difficult to obtain in children.

Isoproterenol and nitroglycerine were the most commonly used pharmacological agents in our survey. Oral nitroglycerine has the advantage of performing the test without having to place an intravenous line. However, a recent study comparing nitroglycerin and isoproterenol-augmented tilt tests in children and adolescents demonstrated that nitroglycerin resulted in more false-positive tests and produced more prolonged vasovagal symptoms [19].

Fasting prior to the test can impact the result of the test [9]. Only 19% of the pediatric EPs recommend a fasting period over 4 hours prior to the test. The shorter fasting duration may be because children may not be able to tolerate longer fasting periods or the potential fear of increased false positive tests with the longer fasting conditions.

These variations in the testing protocols may reflect a lack of conviction on the part of pediatric EPS about the usefulness of tilt table testing. Indeed, a majority of respondents (68%) reported that the results of the tilt table test rarely influenced therapy.

There are several limitations to our study. The response rate to the survey was only 52%. This raises the possibility that only those who felt strongly about tilt table testing may have responded to the questionnaire. Because of the blinded nature of the project, we could not analyze whether there was a difference between the responders and the non-responders with respect to geographic location and the type of practice model. However, in general, all regions and institution sizes appeared to be well represented.

Since this study is a survey of physicians and not an investigation of the test itself, we cannot draw specific conclusions on the utility, efficacy or accuracy of the test based on this data. This study is indicative of the perception of tilt table tests within the pediatric EP community. Further studies to investigate the usefulness of this test are necessary.

## Conclusion

Our survey found that pediatric EPs perceive that tilt table testing is not very useful and that it is being used less frequently in children with syncope. We also found significant variations in the protocol of testing with a clear lack of standardization. Further studies are needed to determine the specific subsets of children with syncope in whom tilt table testing has a role in management. We also see the need for the professional societies caring for children (such as the American Academy of Pediatrics or the Pediatric and Congenital Electrophysiology Society) to come up with a standardized protocol, in the absence of which, this lack of clarity in the field is likely to continue.

## Figures and Tables

**Table 1:**
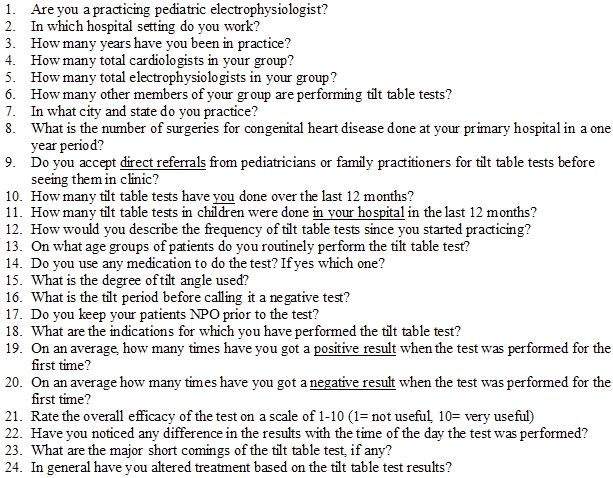
TILT TABLE TEST - Questionnaire

## References

[R1] Benditt DG, Remole S, Milstein S (1992). Syncope: causes, clinical evaluation and current therapy. Annu Rev Med.

[R2] Kapoor WN, Karpf M, Wieand S (1983). A prospective evaluation and follow-up of patients with syncope. N Engl J Med.

[R3] Wayne HH (1961). Syncope: Physiological considerations and an analysis of the clinical characteristics in 510 patients. Am J Med.

[R4] Benditt DG, Ferguson DW, Grubb BP (1996). Tilt table testing for assessing syncope. JACC.

[R5] Abi-Samara F, Maloney JD, Fouad-Tarazi FM (1988). The usefulness of head-up tilt testing and hemodynamic investigations in the work-up of syncope of unknown origin. PACE.

[R6] Almquist A, Goldenberg IF, Milstein S (1989). Provocation of bradycardia and hypotension by isoproterenol and upright posture in patients with unexplained syncope. N Engl J Med.

[R7] Fitzpatrick A, Sutton R (1989). Tilting towards a diagnosis in unexplained syncope. Lancet.

[R8] Kenny RA, Bayliss J, Ingram A (1986). Head up tilt: a useful test for investigating unexplained syncope. Lancet.

[R9] Benditt DG, Ferguson DW, Grubb BP (1996). Tilt table testing for assessing syncope. JACC.

[R10] Alehan D, Celiker A, Ozme S (1996). Head-up tilt test: a highly sensitive, specific test for children with unexplained syncope. Pediatr Cardiol.

[R11] Alehan D, Lenk M, Ozme S (1997). Comparison of sensitivity and specificity of tilt protocols with and without isoproterenol in children with unexplained syncope. Pacing Clin Electrophysiol.

[R12] Strieper MJ, Auld DO, Hulse JE (1994). Evaluation of recurrent pediatric syncope: role of tilt table testing. Pediatrics.

[R13] Boysen A, Lewin MA, Uhlemann F (2006). Common patterns of response to the head-up tilt test in children and adolescents. Cardiol Young.

[R14] Foglia-Manzillo G, Romano M, Corrado G (2002). Reproducibility of asystole during head-up tilt testing in patients with neurally mediated syncope. Europace.

[R15] Kapoor WN, Smith M, Miller NL (1994). Upright tilt testing in evaluating syncope: a comprehensive literature review. Am J Med.

[R16] Lewis DA, Zlotocha J, Henke L (1997). Specificity of head-up tilt testing in adolescents: effect of various degrees of tilt challenge in normal control subjects. J Am Coll Cardiol.

[R17] Natale A, Akhtar M, Jazayeri M (1995). Provocation of hypotension during head-up tilt testing in subjects with no history of syncope or pre-syncope. Circulation.

[R18] Raviele A, Menozzi C, Brignole M (1995). Value of head-up tilt testing potentiated with sublingual nitroglycerine to access the origin of unexplained syncope. Am J Cardiol.

[R19] Vlahos AP, Tzoufi M, Katsouras CS (2007). Provocation of neurocardiogenic syncope during head-up tilt testing in children: comparison between isoproterenol and nitroglycerin. Pediatrics.

